# Protocol for the MS-CEBA study: an observational, prospective cohort study identifying Cognitive, Energetic, Behavioural and Affective (CEBA) profiles in Multiple Sclerosis to guide neuropsychological treatment choice

**DOI:** 10.1186/s12883-024-03737-6

**Published:** 2024-06-28

**Authors:** Anniek Reinhardt, Sandra E. Rakers, Dorothea J. Heersema, Ernesto A. C. Beenakker, Jan F. Meilof, Marieke E. Timmerman, Jacoba M. Spikman

**Affiliations:** 1 Department of Neurology, Neuropsychology Unit, University Medical Centre Groningen, University of Groningen, Hanzeplein 1, Groningen, P.O. Box 30.001, 9700 RB Netherlands; 2 Department of Neurology, University Medical Centre Groningen, Groningen, Netherlands; 3 Department of Neurology, Medical Centre Leeuwarden, Leeuwarden, Netherlands; 4 Department of Neurology and Clinical Neurophysiology, Martini Hospital Groningen, Groningen, Netherlands; 5 Department of Psychometrics and Statistics, University of Groningen, Groningen, Netherlands; 6 Multiple Sclerosis Centre Northern Netherlands, Groningen, Netherlands

**Keywords:** Multiple sclerosis, Neuropsychological profiles, Neuropsychological rehabilitation, Societal participation

## Abstract

**Background:**

Neuropsychological symptoms in the Cognitive, Energetic, Behavioural, and Affective (CEBA) domains are common in people with multiple sclerosis (PwMS) and can negatively affect societal participation. The current study aims to investigate whether there are combinations of symptoms in the different CEBA domains that consistently occur together, that is, if there are CEBA profiles that can be identified. If so, this study aims to develop a screening instrument identifying CEBA profiles in PwMS to select the most suitable neuropsychological rehabilitation treatment for a given CEBA profile and consequently improve the societal participation of PwMS.

**Methods:**

This study is an observational, prospective cohort study consisting of 3 phases. Phase 1 focuses on the identification of CEBA profiles in a large sample of PwMS (*n* = 300). Phase 2 focuses on validating these CEBA profiles through replication of results in a new sample (*n* = 100) and on the development of the screening instrument. Phase 3 focuses on qualitatively evaluating in a small group of PwMS whether the selected treatment is suitable for the given CEBA profile or whether existing neuropsychological treatments should be adapted to meet the needs of PwMS suffering from symptoms in multiple CEBA domains simultaneously. Primary outcome is the CEBA profile, which will be derived from performance on neuropsychological assessment consisting of tests and questionnaires regarding the CEBA domains using a latent profile analysis. Inclusion criteria include MS diagnosis, sufficient ability in the Dutch language, and an age between 18 and 70 years.

**Discussion:**

The results of the current study will contribute to a more comprehensive understanding of the entire spectrum of neuropsychological symptoms in PwMS. Identification of possible CEBA profiles, and accordingly, the development of a screening instrument determining the CEBA profile of PwMS in clinical practice, contributes to the timely referral of PwMS to the most suitable neuropsychological rehabilitation treatment. If necessary, adjustments to existing treatments will be suggested in order to sufficiently meet the needs of PwMS. All of this with the ultimate aim to improve societal participation, and thereby quality of life, of PwMS.

**Trial registration:**

Dutch Central Committee on Research Involving Human Subjects (CCMO) NL83954.042.23; ClinicalTrials.gov NCT06016309.

## Background

Multiple sclerosis (MS) is a disease of the central nervous system characterized by demyelination, inflammation, and neurodegeneration as its main underlying pathologic processes [1]. Approximately 2.8 million people are diagnosed with MS worldwide, and MS is the most common neurological disorder in young adults [2]. The widespread damage to the central nervous system has a variety of physical consequences, such as motor weakness, sensory disturbances and visual problems [3, 4]. In addition to these well-known physical consequences, neuropsychological symptoms are prevalent in MS as well, including cognitive impairments, lack of energy and fatigue, impaired social-behavioural functioning, and affective problems such as anxiety and depression [5–10]. MS usually starts in young adulthood, and at present, there is no cure for the disease [11], underlining the importance of providing people with MS (PwMS) with the best possible support in living with these symptoms.

Both the physical and neuropsychological symptoms of MS can negatively affect societal participation. Societal participation includes the engagement in life situations such as work, social relationships, and leisure activities, which are all crucial for well-being and quality of life [12–17]. To date, the focus in rehabilitation care for PwMS is primarily on physical treatment. Only few PwMS receive access to neuropsychological rehabilitation treatments, although several treatments targeting the neuropsychological symptoms that PwMS present with have been developed, with promising results for societal participation [18–21].

This gap in need for and access to neuropsychological rehabilitation treatment for PwMS could be due to various reasons. It is possible that medical practitioners who see PwMS for regular consultations lack awareness of and knowledge about neuropsychological symptoms and the existing treatments addressing these symptoms. In the already limited time that is usually available in such consultations [22], it is likely that there is a focus on the more visible physical symptoms by patients as well as practitioners [23]. Additionally, in some cases, neuropsychological assessment (NPA) might not occur due to the, by practitioners presumed, burden of undergoing such an assessment. As a consequence, the neuropsychological symptoms of PwMS may go unnoticed for an extended period of time, leaving PwMS to manage these symptoms on their own.

### Neuropsychological symptoms of MS

In the present study, we propose categorizing the neuropsychological symptoms of MS into the Cognitive, Energetic, Behavioural, and Affective (CEBA) domains.

Cognitive impairments are a core symptom of MS, with up to 70% of PwMS presenting with cognitive deficits. The most frequently affected cognitive functions include information processing speed, memory, attention and executive functioning [7, 9, 10]. With respect to the Energetic domain, fatigue is reported by up to 80% of PwMS, with 15–40% even reporting it to be their most debilitating symptom [8, 24–26]. Thus far, the terminology used to describe fatigue in MS varies from pathological fatigue [27] to cognitive fatigue [28] to fatigue in general [26, 29]. Definitions of these terms also vary: although some definitions underscore the different aspects of fatigue, by mentioning both physical and mental components [27], fatigue in MS often seems to be treated as a general concept. With physical as well as nonphysical components, both with different possible effects on societal participation and quality of life [8, 30]. Therefore, the mental and physical components of fatigue will be specifically included as separate constructs in the present study. Concerning the Behavioural domain, PwMS can present with impairments in social cognition [6, 31]. Social cognition involves a range of cognitive abilities that underlie the perception, understanding and reaction to social stimuli, thereby playing a crucial role in our behaviour when interacting with others. Social cognitive impairments frequently lead to behavioural changes which may negatively affect social relationships and social functioning [6, 32–34]. With regard to the Affective domain, PwMS present with higher depression rates than patients with most other chronic neurological diseases [35, 36], ranging from 14 to 54% [5, 37]. Symptoms of anxiety are also common, affecting 14 to 41% of PwMS [5].

Symptoms in all CEBA domains negatively affect social and occupational functioning and thereby societal participation in general, as well as quality of life, of PwMS [6–8, 32–34]. Considering the negative impact of symptoms in one single CEBA domain on societal participation, it seems likely that the co-occurrence of symptoms in multiple CEBA domains simultaneously might have an even greater negative effect on participation. However, this is unknown, since only univariate relationships between symptoms in the CEBA domains and restricted participation have been considered and established thus far [12–14, 16].

### Co-occurrence of symptoms in the different CEBA domains

Several studies have shown that in PwMS, symptoms in the different CEBA domains can occur simultaneously. Depression and anxiety, for example, have been linked to specific cognitive functions, with depression having a negative influence on multiple cognitive domains, including information processing speed, working memory, executive functioning, and attention [38–41], and anxiety primarily affecting information processing speed and nonverbal memory [41, 42]. In addition, depression is known to be related to fatigue [43, 44], and higher levels of self-reported anxiety and depression have been shown to be related to lower performance on tasks for social cognition [45]. Social cognition is correlated with traditional cognitive deficits as well, including attention, executive functioning and memory [6, 46]. Additionally, multiple studies [45, 47] have shown a relationship between social cognitive functioning and fatigue; that is, higher levels of psychosocial fatigue have been associated with inferior performance on tasks for social cognition.

Current neuropsychological rehabilitation treatments primarily focus on symptoms in one CEBA domain at a time. For instance, at present, there is a compensation strategy training targeting cognitive problems [20], a cognitive behavioural therapy focusing on reducing MS-related fatigue [18], a compensatory strategy training focusing on impairments in social cognition and social behaviour [21], and an acceptance and commitment therapy adapted to people with acquired brain injury, targeting anxiety and/or depressive symptoms [19]. Considering the aforementioned co-occurrence of symptoms in the different domains [41, 45], the question arises whether existing neuropsychological rehabilitation treatments sufficiently meet the needs of PwMS or whether these treatments need adaptation to provide appropriate care for PwMS suffering from symptoms in multiple CEBA domains simultaneously.

Up to now, to the best of our knowledge, it has not yet been investigated over the whole spectrum of CEBA domains whether certain combinations of affected domains commonly occur together. In other words, it is unknown whether there are certain CEBA profiles that typically occur among PwMS and, if so, how these combinations of symptoms affect societal participation. By extension, there is currently no standardized procedure for selecting the most suitable neuropsychological rehabilitation treatment for a given CEBA profile, and it is unknown whether the existing treatments adequately address the needs of PwMS with these CEBA profiles. Considering the aforementioned impact of neuropsychological symptoms on societal participation and quality of life, there is currently an unmet need in today’s care for PwMS.

### Objectives

The MS-CEBA study aims to identify possible different CEBA profiles and the relationships between such CEBA profiles and societal participation in PwMS. If different CEBA profiles can be established, the aim is to validate the CEBA profiles found in a new group of PwMS. If successful, our aim is to develop a compact neuropsychological screening instrument that is feasible for use in clinical practice and which allows the timely identification of specific CEBA profiles without putting an unnecessary load on PwMS. With this compact screening instrument, PwMS can be timely referred to the neuropsychological rehabilitation treatment most suitable for their specific CEBA profile. Subsequently, by means of a qualitative evaluation, it will be assessed whether this method of selecting treatment indeed proves effective and the selected treatment is suitable for the given CEBA profile or whether, in future research, the existing neuropsychological treatments should be adapted or combined to meet the needs of PwMS. All of this is done with the overarching aim to improve societal participation and quality of life of PwMS. These objectives and the associated subobjectives are presented in Table 1. The study will be divided into 3 phases, pertaining to [1] identification of the CEBA profiles, [2] validation of the profiles and development of the screening instrument, and [3] evaluation of the recommended neuropsychological rehabilitation treatment.

**Table 1:** Objectives of the MS-CEBA study

***(1) Phase 1: To identify possible CEBA profiles among PwMS***	
To achieve this objective, it will be investigated (1a) which proportion of the PwMS present with which CEBA symptoms, (1b) if PwMS and healthy controls differ on scores on neuropsychological tests and questionnaires regarding the CEBA domains, and if so, on which domain(s) they differ, (1c) which CEBA profiles (i.e., clustering of CEBA symptoms) exist among PwMS, (1d) if CEBA symptoms of PwMS are stable over time, (1e) how the identified CEBA profiles relate with demographic and disease related characteristics, and (1f) to what extent societal participation of PwMS can be predicted by the identified CEBA profiles and/or CEBA symptoms	
***(2) Phase 2: To validate CEBA profiles and develop the CEBA screening instrument***	
To achieve these objectives, it will be investigated (2a) whether the identified CEBA profiles can be replicated in a new sample of PwMS. Subsequently, (2b) a feasible screening instrument allowing timely identification of CEBA profiles among PwMS will be developed, in order to indicate the most suitable neuropsychological rehabilitation treatment	
***(3) Phase 3: To evaluate the recommended neuropsychological rehabilitation treatment***	
To achieve this objective, it will be (3a) qualitatively evaluated in a small group of PwMS from Phase 2, who chose to enter neuropsychological rehabilitation treatment based on the advise of the screening instrument, whether the treatment was adequately matched to their expectations, and by extension it will be (3b) qualitatively evaluated whether adjustments to, or combining of existing neuropsychological rehabilitation treatment options for PwMS with a specific CEBA profile should be suggested for future research	

## Methods

### Study design

The MS-CEBA study is an observational, prospective, cohort study consisting of three phases, as outlined in Table 1. The study aims to include a group of 300 PwMS in Phase 1, of whom 54 will undergo retesting after one year to investigate the stability of the results. Additionally, a group of 100 healthy controls (HCs) will be included in Phase 1. After two years, a second group of 100 PwMS will be included in Phase 2 to investigate whether the results of Phase 1 can be replicated, thereby validating the CEBA profiles. A neuropsychological screening instrument for timely identification of CEBA profiles will then be developed. In Phase 3, a qualitative evaluation of a small group of PwMS (n = 10) from Phase 2 who sought neuropsychological rehabilitation treatment using the advice obtained with the screening instrument (as a part of clinical care, not an intervention in this study) will be performed to identify whether and how this treatment adequately matched their expectations. If necessary, suggestions for adjustments to, or combining of, existing neuropsychological treatments in future research will be made based on this evaluation.

### Study population

#### Criteria

The inclusion criteria for PwMS are a confirmed MS diagnosis, and for HCs the absence of an MS diagnosis. For all, the inclusion criteria are an age between 18 and 70 years, the ability to participate in a short NPA, as judged by the practitioner and/or researcher, and an adequate command of the Dutch language. The exclusion criterion for both PwMS and HCs is the presence of other neurological and/or major psychiatric conditions.

#### Recruitment

PwMS are recruited from the Neurology Unit of the University Medical Centre Groningen (UMCG), Martini Hospital Groningen (MHG) and Medical Centre Leeuwarden (MCL), the Netherlands. PwMS who have been scheduled for regular clinical visits at the outpatient department will be approached by their practitioner and provided with information regarding the study and an informed consent form. In addition, PwMS can independently sign up for participation whenever they hear about the study, e.g., through patient associations. PwMS who sign up for the study themselves will be contacted by the researchers, after which the study information and informed consent form will be sent to their home address. A group of 100 HCs, matched for age and education level, will be recruited through convenience sampling.

### Study procedures

#### Phase 1: Identification of possible CEBA profiles

Data collection and management of the 300 PwMS will be performed by the researchers (neuropsychologists). When informed consent is received, PwMS will be approached by the researcher to schedule the NPA. Prior to the NPA, PwMS will receive questionnaires regarding fatigue, mood, behaviour and societal participation to fill out at home (Table 2). The questionnaires will be sent digitally via the secure web app Research Electronic Data Capture (REDCap) [48, 49] or, upon request, on paper.

**Table 2:** Overview of the assessments used in the MS-CEBA study

Assessment	Construct	Phase 1 PwMS^a^ *(n* = 300)	Phase 1 HCs^b^ (*n* = 100)	Phase 1 Retest PwMS (n = 54)	Phase 2 PwMS (*n* = 100)
**General information (participants)**					
Demographics	Age, sex, education	✓	✓		✓
Medical information	EDSS^c^, disease duration, MS^d^ subtype, MS medication	✓			✓
Anamnesis (structured interview)	Sleep, coping, social network, subjective complaints, highest burden	✓	In part	✓	✓
**Questionnaires (participants)**					
Hospital Anxiety and Depression Scale (HADS)	Anxiety and depression (Affect)	✓		✓	✓
Dutch Multifactor Fatigue Scale (DMFS)	Fatigue (Energy)	✓		✓	✓
Dysexecutive questionnaire (DEX)—self	Dysexecutive syndrome and behavioural changes (Behaviour)	✓		✓	✓
Impact on Participation and Autonomy questionnaire (IPA)	Participation	✓		✓	✓
**Questionnaires (proxies)**					
Dysexecutive questionnaire (DEX)—proxy	Dysexecutive syndrome and behavioural changes (Behaviour)	✓		✓	✓
Behavioural changes questionnaire	Behavioural changes (Behaviour)	✓		✓	✓
**Neuropsychological assessment (participants)**					
Symbol Digit Modalities Test (SDMT)	Information processing speed (Cognition)	✓	✓	✓	✓
15 Words Test (15-WT)	Verbal memory (Cognition)	✓	✓	✓	✓
Digit Span Test of the Wechsler Adult Intelligence Scale (WAIS) IV	Memory span/working memory (Cognition)	✓	✓	✓	✓
Trail Making Test (TMT)	Attentive/executive functioning (Cognition)	✓	✓	✓	✓
Letterfluency Test (LFT)	Attentive/executive functioning (Cognition)	✓	✓	✓	✓
Facial Expressions of Emotion: Stimuli and Tests (FEEST)	Social cognition (Behaviour)	✓	✓	✓	✓
^a^PwMS: People with Multiple Sclerosis; ^b^HCs: Healthy Controls; ^c^EDSS: Expanded Disability Status Scale; ^d^MS: Multiple Sclerosis					

Upon registration for participation, PwMS are asked to provide contact information about a proxy (e.g., significant other or other close relative). Informed consent for the proxy will also be obtained before study participation. The proxy is asked to fill out two questionnaires prior to the NPA appointment, either digitally via REDCap [48, 49] or on paper (Table 2). The proxy does not have to attend the NPA appointment.

The NPA takes approximately 90 minutes and consists of an anamnesis regarding demographic and disease characteristics, sleep, coping style, subjective complaints and highest experienced burden, and a neuropsychological test battery concerning different cognitive domains, including social cognition (Table 2). For the retest of Phase 1, PwMS who at administration for participation gave consent to be approached for possible follow-up research, will be asked to participate in the retest sample, after approximately one year.

From the group of 100 HCs, informed consent will be obtained before study participation as well. The questionnaires and test battery of the NPA will be the same as for the PwMS, but parts of the anamnesis (concerning disease characteristics, subjective complaints, and highest experienced burden), as well as proxy measures, will be excluded.

#### Phase 2: Validation of CEBA profiles and development of the CEBA screening instrument

For Phase 2, a new sample of 100 PwMS will be included following the same procedure for inclusion and assessment as in Phase 1 after approximately two years. Additionally, the development of the CEBA screening instrument will take place. In the event that symptoms in different CEBA domains prevail simultaneously within one CEBA profile, the highest experienced burden of the PwMS in question will be used to help select on which CEBA domain initial neuropsychological rehabilitation treatment should focus. The screening instrument will therefore consist of a concise set of neuropsychological tests and questionnaires identifying combinations of symptoms in the different CEBA domains combined with a short anamnesis regarding the highest experienced burden. Attached will be a manual outlining how to easily derive CEBA profiles from scores on the tests and questionnaires used, along with an advice regarding which neuropsychological rehabilitation treatment would be the most suitable for which combination of CEBA profile and highest experienced burden.

#### Phase 3: Evaluation of the recommended neuropsychological rehabilitation treatment

For the qualitative evaluation of Phase 3, PwMS from Phase 2 who sought neuropsychological treatment using the advice obtained with the screening instrument and who, at administration for participation, gave consent to be approached for possible follow-up research will be asked to share their experience with the recommended treatment in a qualitative evaluation. As a part of this, it will be evaluated whether the proposed method of treatment selection indeed proves appropriate.

### Data collection

#### Primary outcome

The primary outcome of the MS-CEBA study are the CEBA profiles, which are latent and thus need to be derived from the scores on neuropsychological tests and questionnaires used to assess the CEBA domains. Through the use of a latent profile analysis, as described in the *Statistical Analysis* section, CEBA profiles will be revealed based on the raw scores of the tests and questionnaires used.

#### Neuropsychological tests and questionnaires assessing the CEBA domains

Table 2 lists the CEBA domains and the associated neuropsychological tests that will be used. All tests and questionnaires are commonly used in both research and clinical practice within the Netherlands, and either normative data or data from HCs  are available.

#### Secondary outcomes

Secondary outcomes are the relationships of the CEBA profile with demographic and disease-related characteristics, such as MS subtype, age of disease onset, medication and EDSS (obtained from anamnesis or medical records), and level of societal participation (derived from the score on the Impact on Participation and Autonomy (IPA) questionnaire), all of which are listed in Table 2.

### Data management

The data are stored in an electronic Case Report Form (eCRF) using REDCap [48, 49]. The metadata are stored in the eCRF. Study monitoring will be performed by in-house study monitors from the UMCG. Depending on the type of data and associated privacy regulations, data from the MS-CEBA study will be made publicly available or will become available via the corresponding author upon reasonable request.

### Statistical analysis

For analysing the data, Statistical Package for the Social Sciences (SPSS) version 28.0 and/or R, possibly combined with Latent GOLD version 6.0 [50], depending on the availability of suitable packages in R, will be used. For all analyses, the nominal significance level will be set at α = 0.05, 2 sided. The numbering of the analyses described below corresponds to the numbering of the objectives displayed in Table 1*.* In the following, T0 refers to the first group of 300 PwMS, and T1 refers to the retesting of 54 PwMS.

**Phase 1: Identification of possible CEBA profiles.** 1a) Summary statistics will be computed, describing percentages of PwMS (n = 300) with CEBA symptoms, based on scores on the neuropsychological tests and questionnaires regarding each CEBA domain; 1b) MANOVAs with follow-up ANOVAs will be computed comparing PwMS *(n* = 300) to HCs (*n* = 100) on scores on the neuropsychological tests and questionnaires regarding the Cognitive and Behavioural domain, and ANOVAs will be computed comparing PwMS to HCs on scores on questionnaires regarding the Energetic and Affective domain; for 1c) and 1e) a three-step mixture analysis will be computed [51]: step 1 is a latent profile analysis to identify clusters of PwMS with common CEBA profiles; step 2 is to estimate the posterior cluster membership probabilities of the model from step 1 for each PwMS in the sample, and step 3 is to relate the cluster membership probabilities from step 2 to relevant predictors, i.e., a regression analysis will be carried out to estimate the relationship between the CEBA profiles and the demographic and disease-related variables, with an adaptation to account for the fact that cluster membership is estimated, rather than observed; for 1d) repeated measures MANOVAs will be computed comparing T1 (n = 54) to T0 (n = 300) on the scores on neuropsychological tests and questionnaires for the Cognitive and Behavioural domain separately; if a time effect is indicated, these will be followed by post hoc analyses (repeated measures ANOVAs), to assess which variable(s) the time effect pertains to. A repeated measures ANOVA will be computed comparing T1 to T0 on the scores on questionnaires regarding the Energetic and Affective domain separately; 1f) regression analysis will be carried out, considering two competing models: model 1, using the membership of the CEBA profiles as a predictor; and model 2, using the most distinguishing outcome variables (scores on neuropsychological tests and questionnaires in each of the CEBA domains) as predictors.

**Phase 2: Validation of CEBA profiles and development of the CEBA screening instrument.** 2a) The posterior cluster membership probabilities of the selected latent profile model from Phase 1 will be estimated for each of the PwMS in the new sample (n = 100). The equality of the distributions of membership probabilities of the new sample and Phase 1 sample will be tested using a chi-square test. 

In 2b), development of the screening instrument, and in **Phase 3: Evaluation of the recommended neuropsychological rehabilitation treatment**, no statistical analysis will be performed. 

### Sample size

The sample size calculations provided below were performed following G*Power [52]. Since for objective 2b and objectives 3a and 3b (Table 1), no statistical analyses will be carried out, a sample size calculation is not relevant for these objectives.

With regard to objectives 1a and 1c, here, the main study parameter is the CEBA profile, which is latent and thus needs to be derived from performance on neuropsychological tests and questionnaires regarding the CEBA domains. For identification of the CEBA profiles, latent profile analysis will be performed. A minimum sample size of 300 is suggested for latent profile analysis to ensure that there is sufficient power to estimate all model parameters and recover the true number of classes [53, 54]. Therefore, the aim is to include a minimum of 300 patients. For objective 1b, the main study parameters are possible differences between PwMS and HCs on neuropsychological tests and questionnaires regarding symptoms in the different CEBA domains, which will be investigated through the performance of MANOVAs and ANOVAs. A power calculation (with a medium effect size [Cohen’s f] = 0.5, α = 0.05, power = 0.95) results in a minimal sample size of n = 60, which is expected to be exceeded with our goal of including 300 PwMS and 100 HCs for comparison of performance in the CEBA domains. With regard to objective 1d, here, the main study parameters are possible differences in CEBA symptoms between T0 and T1, which are derived from scores on neuropsychological tests and questionnaires regarding the CEBA domains. To investigate the extent to which individual CEBA symptoms are stable over time, repeated measures MANOVAs and ANOVAs will be performed to compare T0 to T1. Within-between interactions will be used. Here, a power calculation using repeated measure analysis (with medium effect size Cohen’s f = 0.5, α = 0.05, and power = 0.95) results in a total sample size of n = 54. Therefore, the aim is to include a minimum of 54 patients for comparing T0 to T1. Since objectives 1e and 1f, as well as objective 2a, depend on the CEBA profiles yet to be identified, the corresponding analyses are considered exploratory, and therefore, no sample size calculation will be provided at this stage. At a later stage, after the CEBA profiles have been identified, sample size calculations for these objectives will be provided, again using G*Power [52].

## Results

Recruitment started in mid-2023 and is planned to continue until the end of 2026. Currently, 167 participants have participated in the study. The first publications are expected in early 2026.

## Discussion

To the best of our knowledge, the MS-CEBA study is the first to investigate, over the whole spectrum of CEBA domains, whether there are symptoms in the different domains that consistently occur together in PwMS. In other words, if certain CEBA profiles can be identified, and in turn, how these possible profiles are related to the level of societal participation of PwMS. We believe that increased awareness of and knowledge on the combinations of neuropsychological problems in MS is a meaningful contribution to the literature and to neuropsychological rehabilitation care for PwMS for various reasons.

First, if specific CEBA profiles are indeed discovered, this is an important step in systematically mapping the neuropsychological symptoms that this heterogeneous patient group presents with. This allows for a more structured screening of these combinations of neuropsychological symptoms in PwMS by developing a time-efficient screening instrument taking into account all CEBA domains as well as the highest experienced burden. Standard deployment of the screening instrument in clinical practice, and thereby more consistent and timely attention to the combinations of neuropsychological symptoms of MS, will help prevent these combinations of symptoms from going unnoticed for an extended period of time. Therefore, in the future, PwMS who present with a given CEBA profile will be more timely and fully informed about the most suitable existing neuropsychological rehabilitation treatment options for their profile, and if desired, treatment can be started in a timely manner.

Second, the current dataset will enable the exploration of demographic and medical data as potential predictors for specific CEBA profiles and, additionally, how each corresponding CEBA profile relates to the level of societal participation. This provides insight into potential risk factors for certain CEBA profiles and the corresponding level of societal participation, contributing further to the timely identification of CEBA profiles and, associated with this, the timely indication for neuropsychological rehabilitation treatment in PwMS.

Third, identifying CEBA profiles and patients’ highest experienced burden when different domains prevail in a profile allows us to examine whether the existing neuropsychological rehabilitation treatment options, currently all targeting a single CEBA domain, adequately address the needs of PwMS with a certain CEBA profile. If, based on the qualitative evaluation in the current study, it turns out that (part of) the existing treatments do not sufficiently align, this serves as a crucial foundation for further research exploring potential adjustments to these existing treatments.

## Conclusions

In conclusion, the results of the MS-CEBA study will contribute to a more comprehensive understanding of the entire spectrum of neuropsychological symptoms in MS and will enable timely recognition of combinations of symptoms in the different CEBA domains (that is, CEBA profiles) in PwMS in clinical practice. This, to ensure that PwMS can be referred to the treatment option most suitable for a given CEBA profile if needed. All of this, with the ultimate aim of maintaining or improving societal participation, and thereby quality of life, of PwMS to the best of our ability.

## Data Availability

Depending on the type of data and the associated privacy regulations, data from the MS-CEBA study will be made available upon reasonable request to the principal investigator (JMS).

## Abbreviations

CEBA: Cognitive, Energetic, Behavioural, Affective
CCMO: Dutch Central Committee on Research Involving Human Subjects
eCRF: Electronic Case Report Form
EDSS: Expanded Disability Status Scale
HCs: Healthy Controls
IPA: Impact on Participation and Autonomy Questionnaire
MCL: Medical Centre Leeuwarden
MHG: Martini Hospital Groningen
MREC: Medical Research Ethics Committee
MS: Multiple Sclerosis
NPA: Neuropsychological Assessment
PwMS: People with Multiple Sclerosis
REDCap: Research Electronic Data Capture
SPSS: Statistical Package for the Social Sciences
UMCG: University Medical Centre Groningen
